# Prevalence of Women Authors in Family Medicine Literature

**DOI:** 10.1001/jamanetworkopen.2019.16029

**Published:** 2019-11-22

**Authors:** Katherine M. Wright, Deborah Edberg, Santina Wheat, Deborah S. Clements

**Affiliations:** 1 Feinberg School of Medicine, Department of Family & Community Medicine, Northwestern University, Chicago, Illinois

## Abstract

This cross-sectional study examines the prevalence of women authors in 5 prominent family medicine journals.

## Introduction

Previous studies demonstrate gender disparities in academic medicine, exemplified by unequal rates of promotion, tenure, early-career attrition, compensation, journal authorship, leadership, and editorial positions.^1,2,3^ A strong publication record is essential to career advancement in academia.^4^ However, as the process of peer review is imperfect and relies heavily on subjective opinions of individuals, this is an area susceptible to bias that deserves attention. In this cross-sectional study, we present empirical explorations of the gender composition of authors in prominent family medicine journals. These data supplement existing literature to provide an update on the trajectory toward gender parity within family medicine research.

## Methods

We conducted a bibliometric analysis of 5 prominent family medicine journals. Journals were selected based on their publication volume among family medicine faculty and include the *Journal of the American Board of Family Medicine*, *Family Medicine*, *American Family Physician*, the *Journal of Family Practice*, and the *Annals of Family Medicine*.^5^ Author names, publication type (eg, journal articles, editorials, case reports), and citation data were abstracted from Ovid MEDLINE for articles published between 2013 and 2017. The policies of Northwestern University’s institutional review board do not require review of this type of study because it did not involve human participants. Reporting of this study follows the relevant requirements of the Strengthening the Reporting of Observational Studies in Epidemiology (STROBE) reporting guideline.

Author gender was derived algorithmically based on first name using the genderizeR package for the R programming language.^6^ We conducted an exact binomial test to compare proportions of women first authors to the proportion of full-time female family medicine faculty reported in annual Association of American Medical Colleges Faculty Rosters.^1^ Results were considered statistically significant at 2-tailed *P* < .05. R statistical software version 3.6.0 (R Project for Statistical Computing) and SPSS statistical software version 25 for Windows (IBM) were used in the analyses.

## Results

A total of 2745 citations were published in family medicine journals and indexed in Ovid MEDLINE during the 5 years included in our review. Of these, 2004 journal articles met our inclusion criteria after excluding editorials, personal narratives, case reports, patient communication handouts, commentaries, and other publication types to narrow our scope to peer-reviewed research articles.

Overall, women composed 44% (95% CI, 42%-46%; *P* < .001) of first authors in our review sample (Figure) despite representing 49% of family medicine faculty. As each journal controls its peer review process, data were disaggregated to determine whether women fare better in specific publications. Women were underrepresented as first authors in the *Journal of Family Practice* at 36% (95% CI, 30%-42%) and in *American Family Physician* at 41% (95% CI, 36%-45%) compared with the benchmark proportion of female family medicine faculty (49%). Women were more equitably represented in the *Journal of the American Board of Family Medicine* (50%; 95% CI, 44%-55%), *Family Medicine* (47%; 95% CI, 43%-52%), and the *Annals of Family Medicine* (44%; 95% CI, 39%-49%).

**Figure.**
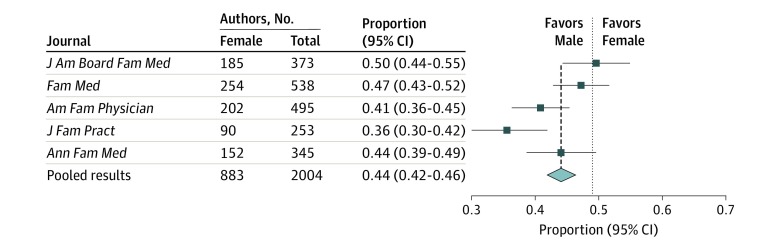
Proportions of Women First Authors by Journal *Am Fam Physician* indicates *American Family Physician*; *Ann Fam Med*, *Annals of Family Medicine*; *Fam Med*, *Family Medicine*; *J Am Board Fam Med*, *Journal of the American Board of Family Medicine*; and *J Fam Pract*, *Journal of Family Practice*.

## Discussion

Representation of women first authors has not yet achieved parity in specific journals. However, the causal mechanisms that underpin these disparities remain unclear. Limitations in the data do not allow us to explore submission rates by gender. The journal with the highest proportion of women first authors, the *Journal of the American Board of Family Medicine*, implements a double-blind peer review process. In contrast, *American Family Physician* requires an author credentialing form listing names, degrees, previous citations, and indicating whether the author published in their journal previously. Further research may be warranted to clarify the impact of gender in peer review processes and to identify interventions to mitigate gender disparities in academic publishing and the broader field of academic medicine.
